# Innovative Variation in the Morphological Characteristics of Carbon Nanowalls Grown on a Molybdenum Disulfide Interlayer

**DOI:** 10.3390/nano12234334

**Published:** 2022-12-06

**Authors:** Chulsoo Kim, Kangmin Kim, Seokhun Kwon, Hyunil Kang, Byungyou Hong, Wonseok Choi

**Affiliations:** 1 Department of Electrical Engineering, Hanbat National University, Daejeon 34158, Republic of Korea; 2 School of Electronic and Electrical Engineering, Sungkyunkwan University, Suwon 16419, Republic of Korea

**Keywords:** carbon nanowall, molybdenum disulfide, interlayer, morphological characteristics

## Abstract

Carbon is a material with interesting properties which exists in large quantities on Earth, so many studies involving carbon have been conducted. In particular, nano-sized carbon allotropes, referred to as carbon nanomaterials, comprise the subject of various studies currently underway. The electrical, chemical, physical properties of carbon nanowalls (CNWs) are modified by parameters such as surface density, height and thickness. These characteristics have significant effects on CNWs and can be adjusted as a growth interlayer. It was confirmed that the molybdenum disulfide (MoS_2_) interlayer synthesized in this paper by radio frequency (RF) magnetron sputtering altered the morphological characteristics of the CNWs, including its shaped edge, pores diameter and density. We provide interesting results through FE-SEM, EDS and Raman analysis in this paper. Based on the Raman analysis, both the D-peak of carbon and the I_D_/I_G_ ratio decreased. Through this study, the effect of MoS_2_ on the morphological characteristics of CNWs was confirmed.

## 1. Introduction

Carbon-based materials have the advantages offered by metals, chemicals and ceramics. They have excellent strength and flexibility, are lightweight and have high electrical conductivity. In addition, carbon-based materials are used in various fields around the world because of the chemical stability they offer [1,2]. Various carbon allotropes exist, depending on hybridization bonding, such as sp^2^, sp^3^, corresponding to graphene, graphite, and diamond. Among them, carbon nanowalls (CNWs), or the vertically oriented structures of graphene [3,4,5,6], are promising candidates with very large specific surface areas [7]. Among carbon allotropes, CNWs can be grown by plasma enhanced chemical vapor deposition (PECVD) at relatively low temperature [8]. Without catalysts, CNWs can be grown on various substrates, such as glass and polymer-based substrates. In addition, CNWs exhibit various physical properties depending to the morphological characteristics. Variations in morphological characteristics depends on several parameters, such as the reactant gas, power and working pressure [9]. These results can be confirmed through diverse literature reports, but studies on morphological characteristics attributed to the interlayer remain limited [10]. However, our research group did not control parameters such as the ratio of the reaction gas, including the microwave power and the working pressure, and the variation in morphological characteristics of the CNWs is brought on by the MoS_2_ interlayer. MoS_2_, one of the representative materials of transition metal dichalcogenides (TMDC), is a two-dimensional material with excellent physicochemical properties [11,12] which has been in the spotlight as a semiconductor material that can replace graphene. Although many studies on carbon-based materials and MoS_2_ hybrid composites have been conducted around the world, there are limitations in that the synthesis is difficult and time-consuming [13]. In this paper, a MoS_2_ interlayer was synthesized on a glass substrate using an radio frequency (RF) magnetron sputtering system to vary the morphological characteristics of CNWs, which shortened the synthesis time and obtained high purity MoS_2_. It was characterized through FE-SEM, EDS and Raman analysis. CNWs grown on the MoS_2_ interlayer showed interesting morphological characteristics. These results are attributed to the initial growth of CNWs in the MoS_2_ crystal plane, and could also be due to van der Waals forces between the surface of MoS_2_ and graphene sheets [14].

## 2. Experimental Method

### 2.1. Preparation of Substrate

A glass substrate consisting of amorphous SiO_2_ was used. In the substrate cleaning process step, ultrasonic degreasing of the glass substrates was performed for 10 min in the following order: trichloroethylene (TCE), acetone, methanol, and deionized water (DI water).

### 2.2. MoS_2_ Interlayer Synthesis and Annealing

The MoS_2_ interlayer was synthesized through an RF magnetron sputtering system using a molybdenum disulfide (MoS_2_, 99.99%) 4-inch target (Table 1). Afterwards, it was annealed in a vacuum chamber at 400 °C and 10^−6^ Torr for 40 min.

### 2.3. Growth of the Carbon Nanowall

The prepared MoS_2_ samples were placed in a PECVD (2.45 GHz microwave) chamber, and a base vacuum at 10^−6^ Torr was applied for 24 h. After 40 sccm of hydrogen (H_2_) gas and 20 sccm of methane (CH_4_) gas were injected into the chamber, a plasma was formed using 1300 W of 2.45 GHz microwave power. During the CNW growth process, the temperature and pressure were maintained at 600 °C and 4 × 10^−2^ Torr, respectively (Table 2).

### 2.4. Characterization and Analysis of Materials

In this study, the morphological characteristics of CNWs were analyzed through field-emission scanning electron microscopy (FE-SEM, HITACH, Japan, S-4800) at 15 kV and energy-dispersive X-ray spectroscopy (EDS). In addition, the intrinsic properties of carbon, such as the graphitization degree and defects of CNW were analyzed using Raman spectroscopy (HORIBA, Japan, LabRAM HR-800). The laser power was 3 mW, the excitation wavelength was 532 nm and a ×50 objective with NA = 0.5 was used.

## 3. Results and Discussion

### 3.1. Molphological Characteristics of Carbon Nanowalls

**Figure 1:**
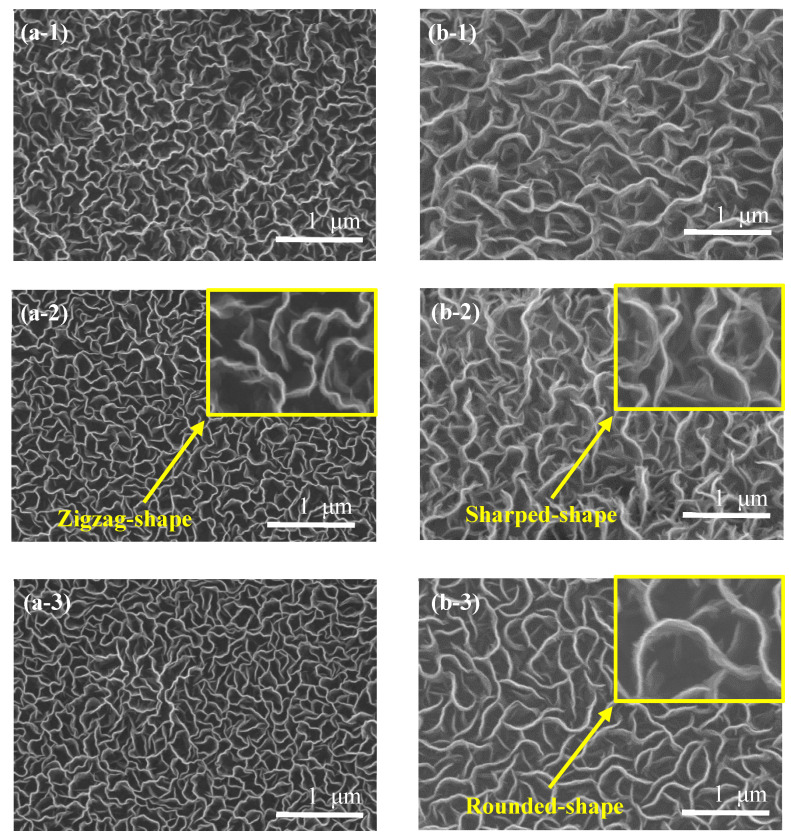
FE-SEM surface images of grown CNWs on the glass substrate with a growth time of (**a-1**) 5 min, (**a-2**) 10 min and (**a-3**) 15 min. Samples (**b-1**–**b-3**) are FE-SEM surface images of CNWs grown on the MoS_2_ interlayer for 5 min, 10 min and 15 min, respectively.

**Figure 2:**
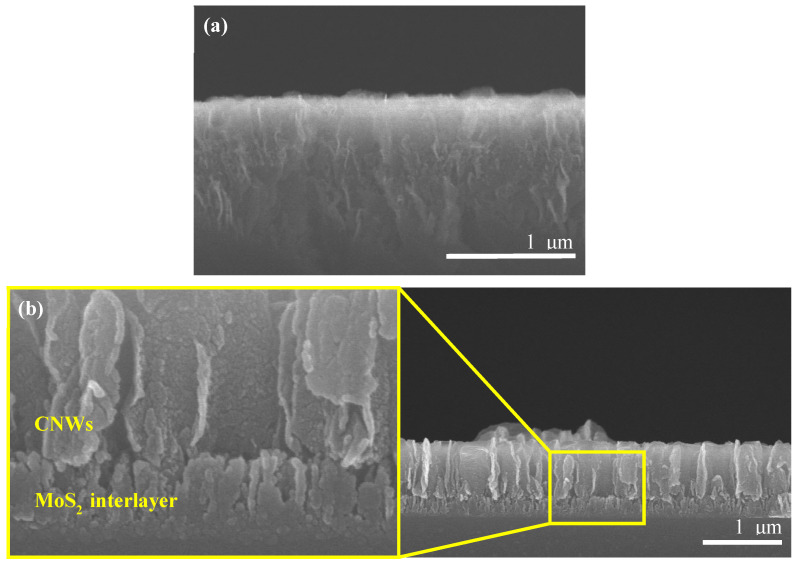
Cross-sectional FE-SEM image of a CNW: (**a**) CNW grown for 10 min on a glass substrate; (**b**) CNW grown for 10 min on the MoS_2_ interlayer and the corresponding magnified image.

**Figure 3:**
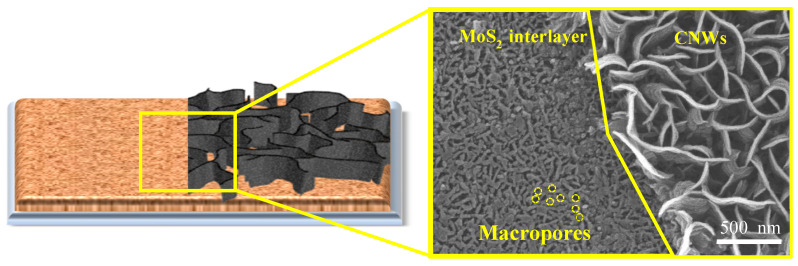
Schematic illustration and corresponding FE-SEM surface images including MoS_2_, the CNW and the interface.

Figure 1(a-1–a-3) shows the surface FE-SEM image of the CNW grown on a glass substrate. The CNW surface is serpentine and disordered regardless of the growth time. It exhibits morphological characteristics of primitive CNWs and is defined by the zigzag-shaped edge [15], shown in Figure 1(a-2). This is because CNW grows anisotropically or is disordered due to defects in the graphene sheets that occur during the initial growth process [16]. Figure 1(b-1–b-3) shows the surface FE-SEM image of CNW growth on the MoS_2_ interlayer. In this case, Figure 1(b-1,b-2) shows sharp-edge shapes [17,18] compared to CNWs grown directly on the glass substrate. As the deposition time was increased to 15 min, a round-edged shape formed in Figure 1(b-3), and compared to Figure 1(a-1–a-3), the density was lower and the pore diameter between the edges increased. The CNW grown on the MoS_2_ interlayer for 10 min showed the greatest deformation in surface morphological characteristics of the existing CNW.

**Figure 4:**
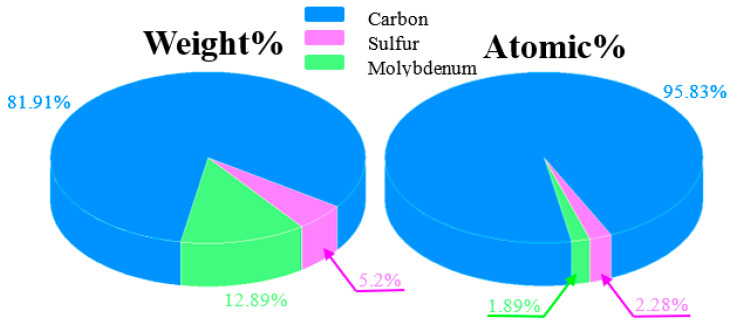
EDS component analysis of the CNW/MoS_2_ sample.

Cross-sectional FE-SEM images of CNW/MoS_2_ samples synthesized on glass substrates for 10 min each are shown in Figure 2. The height is 250 nm for MoS_2_ and 750 nm for CNWs. The vertically oriented MoS_2_ sheet and graphene sheet regions have different diameters and densities. For this reason, we show a clear interface between MoS_2_ and the CNW. They grow at a slower rate than native CNWs because of the initial growth process of CNW occurring in macropores (<50 nm) in the MoS_2_ interlayer (Figure 3). For a 10 min synthesis, CNW grown directly on a glass substrate grew to a height of about 1 μm, whereas the MoS_2_ interlayer grew to 750 nm The morphological characteristics of CNW vary under the influence of the MoS_2_ interlayer, and this variation can be attributed to the interaction between sulfur or molybdenum atoms on the MoS_2_ surface and carbon atoms in the graphene sheet. A graphene sheet with a low defect density is formed in the growth of the initial process by van der Waals forces generated at the interface, and it grows with a sharp-shaped edge. A stoichiometric analysis of the CNW/MoS_2_ sample by EDS was performed and is shown in Figure 4. This result confirmed the existence of CNWs and MoS_2_, and also confirmed that the sample was successfully synthesized.

### 3.2. Raman Spectra

**Figure 5:**
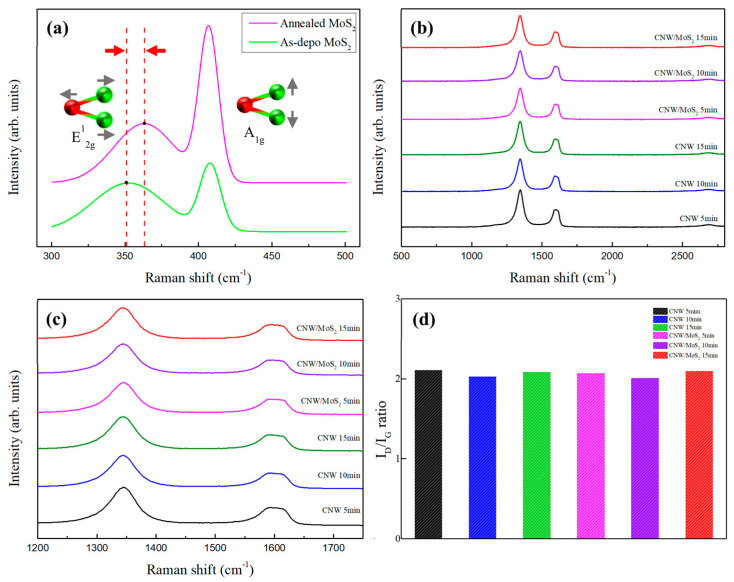
Raman spectra of CNWs and MoS_2_: (**a**) Raman spectroscopic analysis results before and after annealing of the MoS_2_ interlayer; (**b**) Raman spectroscopic analysis results of each CNW sample grown under various conditions and (**c**) the corresponding highresolution Raman spectrum with D and G peaks enlarged; and (**d**) I_D_/I_G_ ratio of each CNW sample grown under various conditions.

Figure 5a shows the results of the Raman spectroscopy analysis before and after annealing of the MoS_2_ interlayer. E^1^_2g_, showing in-plane vibrational characteristics, and A_1g_, showing interlayer vibrational characteristics, were observed. Compared to pristine MoS_2_, annealed MoS_2_ exhibited a peak shift of approximately 11 cm^−1^ in the E^1^_2g_ mode from 351 cm^−1^ to 362 cm^−1^ [19]. The blue shift phenomenon is due to the increase in the van der Waals forces between the MoS_2_ interlayers during the annealing process. When the thickness of the interlayer decreases, the distance between E^1^_2g_ and A_1g_ decreases [20]. Results of the Raman spectrum analysis of the CNW are shown in Figure 5b. Defects in graphite or amorphous carbon cause a high intensity D-peak, and this was observed at 1345 cm^−1^ [21]. The presence of the MoS_2_ interlayer decreases the D-peak of CNW (Figure 5c). In addition, there is a G-peak at about 1592 cm^−1^, which is commonly found in carbon-based materials, and appears due to sp^2^ bonding or the effect of graphitization [22]. The peak observed at around 2686 cm^−1^ is a 2D peak, indicating the double resonance of the pi-bond. When the number of layers of graphene is relatively low, the relatively higher intensity peaks appear. The CNW exhibits low-intensity peaks due to the existence of multi-layer graphene [23].

## 4. Conclusions

In summary, MoS_2_, a transition metal dichalcogenide material, was successfully synthesized using the RF magnetron sputtering system, while CNW, a carbon allotrope, was grown using the PECVD method. MoS_2_ was used as an interlayer material, represents the key subject of this study. The surface density, pores diameter, and growth rate of CNW were changed, and results were characterized through SEM and EDS. Based on these results, it was confirmed that the MoS_2_ interlayer is an innovative material that greatly affects the initial growth of CNW and consequently causes variation in its morphological characteristics. In addition, further studies have shown the possibility of application to various nanostructure growth.

## Figures and Tables

**Table 1:** Sputtering system parameters for MoS_2_ interlayer synthesis.

Parameters	MoS_2_
RF Power	200 W
Base Pressure	10^−6^ Torr
Working Pressure	1.5 × 10^−2^ Torr
Temperature	Room Temperature
Synthesis Time	10 min

**Table 2:** Process parameters of PECVD method for CNW growth.

Parameters	CNW
Microwave Power	1300 W
Reaction Gas	H_2_ 40 sccm and CH_4_ 20 sccm
Base pressure	10^−6^ Torr
Working Pressure	4 × 10^−2^ Torr
Temperature	600 °C
Growth Time	5, 10 and 15 min

## Data Availability

The data presented in this study are available in article.
